# New policy for ICU visits: prospective study

**DOI:** 10.1186/cc13213

**Published:** 2014-03-17

**Authors:** A Moughabghab, S Gemayel, N Azar, R Bousaba

**Affiliations:** 1 Lebanese Canadian Hospital, Beyrouth, Lebanon

## Introduction

ICU visits are generally limited to 2 hours per day selected at any time during the 24 hours. At our institution we abide to the international regulations which were recently modified allowing family members and significant others to be present throughout the day accompanying their ICU-admitted relatives, participating in an uninterrupted timely fashion and no time limits with their daily activities.

## Methods

From January 2012 through April 2012, 200 questionnaires were filled by families of ICU-admitted patients; each questionnaire includes 13 questions concerning their overall degree of satisfaction at our unit and evaluating the quality. Accommodating spaces were offered for families of ICU patients, offering them a state of freedom within the ICU premises surrounding their beloved sick relatives; additionally they were constantly advised by a designated secretary to share their experience and any possible insight to improve the overall polity of our service.

## Results

Two hundred patients were included in the study, the average age of patients was 68.55 years and the average length of ICU stay was 5.2 days. Most family (85%) desired their presence accompanying their related patients within the ICU premises when meals were served. Those families expressed satisfaction while offering psychological support to their beloved ill ones. Fifteen per cent of the families argued about the permitted number of visitors (one person) per visit and 12% addressed lack of communication between the medical staff and patient's families.

## Conclusion

Compared with the limited visit status within the ICU ward in the past, the majority of families expressed general acceptance and satisfaction toward the newly adopted policy. Additionally and by this new policy, patients displayed marked improvement at the psychological level, being more cooperative and experiencing less episodes of agitation. Even the medical team elaborated their interest towards this policy as being a more profound and comprehensive way of supporting the ICU patients. Further investigations are currently being employed to strengthen the outcome of the study.
